# Intact and middle‐down CIEF of commercial therapeutic monoclonal antibody products under non‐denaturing conditions

**DOI:** 10.1002/elps.202000013

**Published:** 2020-04-27

**Authors:** Johannes Schmailzl, Marcel W. Vorage, Hanno Stutz

**Affiliations:** ^1^ Department of Biosciences University of Salzburg Salzburg Austria; ^2^ Christian Doppler Laboratory for Innovative Tools in the Characterization of Biosimilars Salzburg Austria

**Keywords:** adalimumab, CIEF, intact and middle‐down, lysine variants, rituximab

## Abstract

A two‐step CIEF with chemical mobilization was developed for charge profiling of the therapeutic mAb rituximab under non‐denaturing separation conditions. CIEF of the intact mAb was combined with a middle‐down approach analyzing Fc/2 and F(ab´)_2_ fragments after digest with a commercial cysteine protease (IdeS). CIEF methods were optimized separately for the intact mAb and its fragments due to their divergent p*I*s. Best resolution was achieved by combining Pharmalyte (PL) 8–10.5 with PL 3–10 for variants of intact rituximab and of F(ab´)_2_ fragments, respectively, whereas PL 6.7–7.7 in combination with PL 3–10 was used for Fc/2 variants. Charge heterogeneity in Fc/2 dominates over F(ab´)_2_. In addition, a copy product of rituximab, and adalimumab were analyzed. Both mAbs contain additional alkaline C‐terminal lysine variants as confirmed by digest with carboxypeptidase B. The optimized CIEF methods for intact mAb and Fc/2 were tested for their potential as platform approaches for these mAbs. The CIEF method for Fc/2 was slightly adapted in this process. The p*I* values for major intact mAb variants were determined by adjacent p*I* markers resulting in 9.29 (rituximab) and 8.42 (adalimumab). In total, seven to eight charge variants could be distinguished for intact adalimumab and rituximab, respectively.

**Abbreviations**
CA: carrier ampholyte
CEX: cation exchange chromatography
CI: confidence interval
CPB: carboxypeptidase B
IDA: iminodiacetic acid
IdeS: IgG‐degrading enzyme of *Streptococcus pyogenes*
PL: pharmalyte
QA: quality attribute
t_m_: CIEF analysis time (including focusing and mobilization)

## 1 Introduction

Biopharmaceuticals/biologics take a share of 20% in the portfolio of the pharmaceutical industry with progressive growth [1]. Among the top 10 selling drugs worldwide, six products were antibody‐based therapeutics including an Fc‐fusion protein and mAbs in 2017. The latter include adalimumab and rituximab [2]. The mAb market is expected to double until 2022 [3]. The elaborate manufacturing of mAbs currently restricts their wide‐range application due to the resulting costs for public health systems [4]. However, with the expiry of their patent protection follow‐on products, so‐called biosimilars, can be launched [5]. Biosimilars are expected to cover 4% of the entire biopharmaceutical sales within the next decade [6] and to reduce prices that enhances the accessibility of these therapies [7, 8]. Ideally, biosimilars have to be comparable to their reference product in multiple aspects to ensure equivalent efficacy and safety [4]. Due to their molecular structure and the complex manufacturing procedure, clinically irrelevant differences from the genuine reference product are possible [5]. Thus, biosimilar products are similar in all relevant aspects rather than identical to their reference. This biosimilarity is compared with a totality of evidence concept [9] by addressing and comparing quality attributes (QAs) [5], which include inter alia charge variants of the target product. For this purpose, QAs are addressed by a set of complementary analytical methods, respectively.

Different CE modes are used to characterize biopharmaceuticals, including CGE, CZE, and CIEF [10, 11, 12]. For mAbs, preferably CZE and CIEF are applied [13, 14, 15, 16, 17]. For charge variants, CIEF offers a complementary approach to cation exchange chromatography (CEX), which is currently widely used in industry in this context [18]. Contrary to CEX, CIEF can be optimized in a more straightforward way, it shows low volume consumption of separation media and the sample, and omits interactions with the stationary phase [19]. Moreover, CIEF offers the highest selectivity of all CE modes [20]. This is due to a separation of analytes according to their respective p*I* within a pH gradient. The principle of CIEF has been reviewed comprehensively elsewhere [21, 22, 23]. In brief, a pH gradient is formed by an acidic anolyte and an alkaline catholyte [24], which is stabilized by amphoteric compounds, so‐called carrier ampholytes (CAs). CIEF is nowadays performed via a two‐step approach in coated capillaries with suppressed EOF, whereby analytes are first focused and then mobilized toward the detector. The p*I*‐based resolution in CIEF is outlined below

(1) $$\Delta pI\;=\;3\times \sqrt{\frac{D\left(\frac{d\mathrm{pH}}{dx}\right)}{E\left(\frac{-d\mu }{d\mathrm{pH}}\right)}},$$

where Δp*I* is the resolvable p*I* difference of adjacent analytes, D is the analyte diffusion coefficient, E is the electric field strength, *d*pH/*dx* is the slope of the pH gradient, and –*d*μ/dpH is the mobility change with pH [25]. Different mobilization approaches of focused analyte zones have been applied, including hydraulic and chemical mobilization [20, 26]. In general, chemical mobilization with zwitterions provided best resolution [26]. Although the separation profile in CIEF is theoretically considered timely stable, ITP‐based drift processes have been described causing the most acidic and/or alkaline CA species to leave the capillary. This changes the slope of the pH gradient and thus the focusing position of analytes [27, 28]. To combat this effect, so‐called spacer compounds with a p*I* between the most acidic CA and the anolyte and the most alkaline CA and the catholyte are applied, respectively [29]. These compounds should possess properties of good CAs and prevent the addressed loss of CAs and analytes. Moreover, spacers have been applied to block the capillary section behind the detection window thus preventing analyte focusing in the dead‐end capillary section [26, 30]. CIEF has been applied for instance in the analysis of recombinant proteins [26, 31, 32], hemoglobin variants [33, 34], and immune complexes [35]. Nowadays, CIEF is progressively applied in the characterization of biopharmaceuticals including antibodies [13, 36, 37, 38, 39, 40, 41].

Biologics, such as mAbs, play an important role in the medical therapy of numerous diseases. Rituximab is a chimeric antibody of IgG1κ isotype with murine variable domains and human constant domains [42, 43]. It binds to the CD20 antigen of (pre)mature B‐cells and is therefore applied in the therapeutic treatment of B‐cell related tumors, e.g., non‐Hodgkin‐lymphoma, and in autoimmune disorders [44, 45]. Rituximab has the second highest global sales number of mAbs, accounting for 7.5 billion US$ in 2017 [2, 42]. It possesses the most alkaline p*I* among the therapeutic mAbs [46], which impedes CE‐based separations due to the enhanced adhesion onto separation capillaries [47]. In CZE, this was counteracted by BGEs of high ionic strength, dynamic coating additives, and neutral detergents [47, 48, 49]. This work targets to optimize CIEF methods for the reference product of rituximab, i.e., MabThera^®^, combining to our knowledge for the first time CIEF data from intact mAb and a CIEF middle‐down approach after digest with the IgG‐degrading enzyme of *Streptococcus pyogenes* (IdeS). The focus is on the distinction of charge variants based on their different p*I*s. CIEF separations are done under non‐denaturing conditions. The optimized CIEF methods were also applied to a copy product of MabThera^®^, i.e., Reditux^TM^, and the top‐selling mAb adalimumab (Humira^®^) to test their potential as platform methods.

## 2 Materials and methods

### 2.1 Capillary electrophoresis

CIEF measurements were done with an Agilent 7100 CE System (Agilent Technologies, Waldbronn, Germany) with an integrated diode‐array detector (190–600 nm). A 280 nm high pass detector filter assembly from Agilent Technologies was inserted in the light path. Detection was done at 280 nm (4 nm bandwidth) with a reference wavelength of 360 nm (100 nm bandwidth) and a scan rate of 2.5 Hz using an alignment interface for 50 μm straight capillaries. The scan rate was adjusted to the average peak width in order to assure 30 data points per peak. Together with the settings of the reference wavelength, this assures a reduction in the baseline noise. Data acquisition and treatment was done with ChemStation, Rev. B.04.03(16) from Agilent. CIEF separations were performed in eCAP^TM^ CIEF capillaries with neutral coating (Sciex, Framingham, MA, USA) with 50 μm id, 365 μm od, an effective capillary length to detector (L_D_) of 24.2 ± 0.2 cm and a total capillary length (L_T_) of 32.2 ± 0.2 cm. CIEF separations were run at 25.0°C. A current limit of 25 μA was programmed to limit the stress for the capillary coating.

### 2.2 Chemicals

NH_4_HCO_3_ (LC–MS grade), glycine (≥99%; HPLC quality), iminodiacetic acid (IDA; 98%), l‐arginine (l‐Arg; ≥99.5%, BioUltra), l‐aspartic acid (l‐Asp), l‐glutamic acid (l‐Glu), and l‐histidine (l‐His; all ≥99.5%, in p.A. quality) were from Sigma–Aldrich (St. Louis, MO, USA). Tris(hydroxymethyl)aminomethane (min. 99%) was from Serva (Heidelberg, Germany). NaOH (1 M) was from AppliChem GmbH, 85% (m/v) *ortho*‐phosphoric acid and 32% NaOH were purchased from Merck (all Darmstadt, Germany). IdeS, a cysteine protease commercialized as FabRICATOR^®^, was from Genovis (Lund, Sweden) and purchased in form of the FragIT kit, which contains a spin column with immobilized IdeS and a purification column (Capture select) with an affinity ligand for Fc fragments. Carboxypeptidase B (CPB) solution (5 mg/mL) from porcine pancreas was purchased from Roche (Basel, Switzerland). Pharmalyte 3–10 (PL 3–10), PL 8–10.5, and PL 6.7–7.7 (all with 36% m/v) were from GE Healthcare Bio‐Sciences AB (Waukesha, WI, USA). cIEF gel was purchased from Sciex. Markers with p*I* of 9.99, 9.50, and 7.00 were from Sciex, whereas the marker with p*I* 8.40 was kindly provided by Advanced Electrophoresis Solutions (AES) Ltd. (Cambridge, ON, Canada). Tailored peptidic p*I* markers, i.e., Trp‐His‐His‐His‐Asp‐Lys (p*I* 7.56) and Trp‐His‐His‐His‐Glu (p*I* 6.77) were synthesized in‐house in a purity ≥94%. Their identity was confirmed by MALDI–TOF‐MS. Ultrapure water was supplied by a Milli‐Q Plus 185 system (Millipore S.A., Molsheim, France).

### 2.3 Monoclonal antibodies

MabThera^®^ (rituximab reference product) was from F. Hoffmann‐La Roche AG (Basel, Switzerland) and provided as a 10.0 mg/mL aqueous solution containing sodium citrate dihydrate, sodium chloride, and polysorbate 80, at pH 6.5. Reditux^TM^ (10.0 mg/mL; copy product) was from Dr. Reddy´s Laboratories Ltd. (Hyderabad, India) and provided in the same formulation buffer as MabThera^®^. Humira^®^ (adalimumab) drug product (48.5 mg/mL, pH 5.2) was from AbbVie Inc. (Lake Bluff, Il, USA), containing mannitol, citric acid monohydrate, sodium citrate, sodium dihydrogen phosphate dihydrate, disodium phosphate dihydrate, sodium chloride, polysorbate 80, and sodium hydroxide. All antibodies were stored below −60°C.

### 2.4 Antibody digest for middle‐down CIEF

Antibodies were digested with IdeS, which cleaves at a defined sequence C‐terminal to the hinge region thus providing F(ab´)_2_ and Fc/2 fragments [40]. Further details are given in the Supporting Information. Final concentrations of F(ab´)_2_ and Fc/2 fragments prepared in ultrapure water were determined, respectively, by means of an UV nano‐spectrophotometer, i.e., Nanodrop P 330, from Implen GmbH (Munich, Germany).

### 2.5 Digest of Fc/2 fragments with carboxypeptidase B

C‐terminal Lys residues were cleaved from Fc/2 fragments by digest with CPB. Therefore, 6.0 μL of the Fc/2 fraction (0.5 mg/mL in ultrapure water) and 0.12 μL of the commercial CPB solution were mixed and incubated at room temperature for 30 min. The digest preparation was mixed every 5 min. Subsequently, the CIEF sample was prepared from the CPB digest and injected 60 min after the digest initiation.

### 2.6 Capillary isoelectric focusing

Conditioning, rinsing, and storage conditions of the eCAP^TM^ CIEF capillaries were done as described [26]. Details are outlined in the Supporting Information. A total of 500 mmol/L l‐Arg and 100 mmol/L IDA were prepared in ultrapure water, respectively, serving as stock solutions of spacer compounds. A total of 200 mmol/L H_3_PO_4_ (in cIEF gel) and 300 mmol/L NaOH were used as anolyte and catholyte, respectively, and prepared freshly every day. For chemical mobilization, a 25 mmol/L l‐Asp solution was prepared in ultrapure water and adjusted to pH 10.50 with 32% NaOH. This solution was filtered through a 0.45 μm syringe filter and could be used for 2 weeks when stored at +4°C. The sample composition depends on the analyte and is outlined in the text. Anolyte, catholyte, mobilizer solutions, and CIEF samples were vortexed and centrifuged at 14 000 × *g* for 5.0 min at +4°C to remove particulate matter and air bubbles.

The sample was injected for 200 s with 930 mbar to ensure whole‐capillary filling. After the sample injection, the capillary ends were shortly dipped in ultrapure water to avoid carryover effects. Focusing was done at +25.0 kV with the outlined anolyte and catholyte solutions (anolyte at the capillary inlet). Chemical mobilization was performed at +30.0 kV after the catholyte was replaced by the mobilization solution. Anolyte and catholyte were replenished every four runs to avoid depletion effects. The duration of the focusing and mobilization step depends on the analytes and the applied CA combination, which was optimized for the respective analytes. Details are specified in the text.

## 3 Results and discussion

The alkaline p*I* of rituximab and its high molecular mass constitutes a challenge for CIEF. This is due to the paucity of good, i.e., well‐focusing, CA species in the alkaline domain of commercial products [50] and the pronounced adhesion of large alkaline proteins onto capillaries with incompletely masked silanol groups [51]. Typically, denaturing agents, e.g., urea [13, 16, 37] or detergents [52], are added to protein samples in CIEF to minimize adsorption and prevent protein aggregation and precipitation. As protein unfolding biases p*I* values in comparison to the native state and might mask differences between variants, CIEF was performed under non‐denaturing conditions.

### 3.1 CIEF of intact rituximab

**Figure 1:**
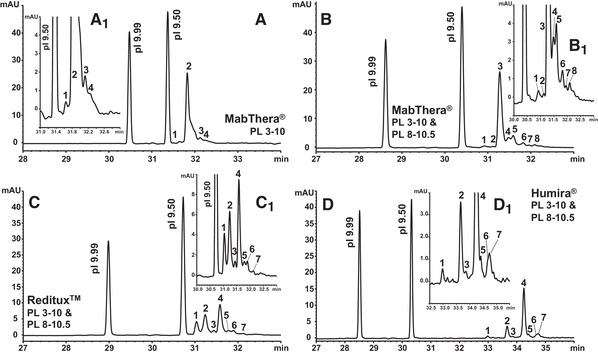
CIEF separation of intact therapeutic monoclonal antibodies. (A) 1.29% (m/v) PL 3–10; (B–D) 1.29% (m/v) PL 3–10 and 0.51% (m/v) PL 8–10.5. Marker: p*I* 9.99 and 9.50. Spacer: 42.9 mmol/L L‐Arg, 0.9 mmol/L IDA. Intact mAbs: (A, B) MabThera^®^, (C) Reditux^TM^, (D) Humira^®^, all with 17.9 μg/mL. All samples contain cIEF gel. Anolyte: 200 mmol/L H_3_PO_4_ (in cIEF gel), catholyte: 300 mmol/L NaOH. Focusing: 25.0 kV, 20.0 min. Chemical mobilization: 25.0 mmoL/L L‐Asp (cathodic), pH 10.50; 30.0 kV. Capillary: eCAP (Sciex) 50 μm id, 365 μm od, L_D_ 24.3 cm, L_T_ 32.3 cm. 280 nm. Acidic and alkaline variants are assigned in relation to the major peak. Peaks are numbered in their order of occurrence. (A_1_)–(D_1_) provide details.

For improved CIEF resolution the sectoral slope of the pH gradient, which covers the focusing site of analytes, should be shallow (see equation 1). A p*I* of 9.26 ± 0.04 (mean ± 95% confidence interval [CI] ) was determined experimentally for intact MabThera^®^ in PL 3–10 by using two markers with p*I* 9.99 and p*I* 9.50 (Fig. 1A). This corresponds with previously published p*I* values [46]. Thus, a narrow pH range CA, i.e., PL 8–10.5, was applied in combination with PL 3–10. The implementation of PL 3–10 allows to address variants and/or impurities with p*I*s not covered by PL 8–10.5. For an optimization of the resolution of intact rituximab variants, the content of PL 8–10.5 was increased stepwise from 0.26 to 0.76% (m/v) while keeping PL 3–10 at 1.29% (m/v). 0.51% (m/v) PL 8–10.5 provided the best resolution (Fig. 1B). A cathodic spacer concentration of 42.9 mmol/L l‐Arg was required to prevent rituximab from migrating in the detection window during the focusing step. The high p*I* of rituximab excludes anodic analyte losses. Thus, only 0.90 mmol/L IDA were included in the sample to prevent losses of acidic CAs. The optimized combination of 0.51% (m/v) PL 8–10.5 with 1.29% (m/v) PL 3–10 resolved two alkaline and five acidic variants from the rituximab main variant (Fig. 1B, B_1_). With exclusive application of PL 3–10, only one alkaline and two acidic signals were separated from the rituximab main peak (Fig. 1A, A_1_). The shoulder at the acidic side of the major peak (Fig. 1A) is apparently caused by the two prominent acidic variants, which are separated in the presence of PL 8–10.5 (peaks 4 and 5, Fig. 1B, B_1_). This is due to the increased occupation of the separation capillary by PL 8–10.5 that selectively flattens the slope of the pH gradient in the focusing domain of the analyte.

#### 3.1.1 Chemical mobilization of intact rituximab

**Figure 2:**
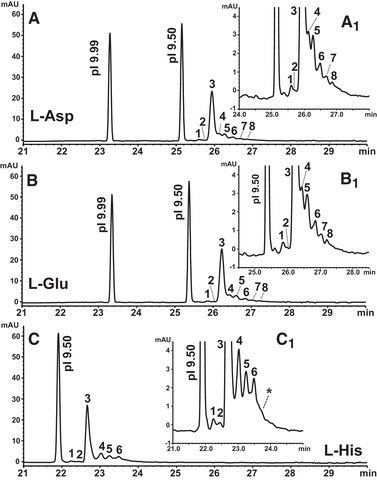
Optimization of cathodic mobilization with zwitterions. MabThera^®^ (intact): 17.9 μg/mL. 1.29% (m/v) PL 3–10 with 0.51% (m/v) PL 8–10.5. Marker: p*I* 9.99 and 9.50. Focusing: 25.0 kV, 20.0 min. Chemical mobilization (cathodic) with (A) 25.0 mmol/L L‐Asp, (B) 25.0 mmol/L L‐Glu, (C) 25.0 mmol/L L‐His, all adjusted to pH 10.50; 30.0 kV. All other settings as in Figure 1. (A_1_)–(C_1_) provide details. “*” in (C_1_) indicates a shoulder most likely containing acidic variants resolved in (A_1_) and (B_1_).

Chemical mobilization provides a considerably improved preservation of the focused profile compared to hydraulic mobilization [26, 53]. Thus, the catholyte was exchanged against zwitterion solutions, i.e., Asp (p*I* 2.77), Glu (p*I* 3.22), or His (p*I* 7.47), for cathodic mobilization. These amino acids are qualified as good CAs that ensures their focusing in distinct zones [54]. Solutions of zwitterions were tested at 25 mmol/L and adjusted to pH 10.50 with NaOH. At this pH the tested amino acids possess a negative net charge in the cathode vessel. Thus, they migrate into the capillary and within the pH gradient. Since their p*I* is more acidic than that of the mAb, the tested amino acids migrate through the focused analyte zones. This way, focused analytes are mobilized (i) by acquisition of a positive net charge in response to the disruption of the pH gradient caused by the invading zwitterions, and (ii) by a growth of the focused mobilizer zone [20, 30]. The optimized CA combination of the previous section was maintained when comparing zwitterionic mobilizers. By mobilization with Asp and Glu two minor alkaline variants and five acidic variants were resolved from the major MabThera^®^ peak (Fig. 2A and B). With His, two alkaline variants, but only three acidic variants were resolved. Apparently, the missing acidic minor variants occurred as a shoulder (see “*” in Fig. 2C_1_). Since Asp provided slightly faster t_m_ than Glu (Fig. 2A and B), mobilization with 25 mmol/L Asp was selected for further experiments.

#### 3.1.2 CIEF platform approach for intact Reditux^TM^ and Humira^®^

The CIEF method with 1.29% (m/v) PL 3–10 and 0.51% (m/v) PL 8–10.5 was also applied for the analysis of intact Reditux^TM^ and Humira^®^, whereby the p*I* markers were kept for comparability reasons. In comparison to MabThera^®^, two prominent alkaline variants (with p*I* 9.41 and 9.35, respectively; see Supporting Information Table S1) were encountered for intact Reditux^TM^ (peaks 1 and 2 in Fig. 1C). The major peak of Humira^®^ occurred at a considerably lower p*I* of 8.42 (Supporting Information Table S1). This is 0.3–0.5 p*I* units smaller than values reported in literature, but there distant markers (i.e., p*I* 4.05 and 10.17) were applied [46] or denaturation with urea was used [55]. The major peak of Humira^®^ is flanked by three alkaline and three acidic variants (Fig. 1D).

### 3.2 CIEF middle‐down approach for rituximab

Subsequently, fragments derived from an IdeS digest were analyzed in order to relate separation profiles of middle‐down to intact analysis. For both, F(ab´)_2_ and Fc/2 fragments, a preliminary calculation of their respective p*I* was done by means of p*I* markers (data not shown). Since F(ab´)_2_ and Fc/2 fragments differed by more than one p*I* unit, the CIEF optimization was performed separately for either fragment.

#### 3.2.1 CIEF optimization for F(ab´)_2_ variants

**Figure 3:**
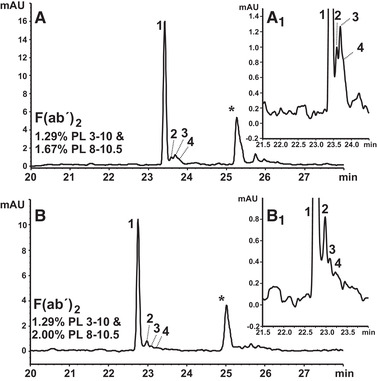
CIEF optimization for variants of F(ab´)_2_ fragment derived from MabThera^®^. 1.29% (m/v) PL 3–10 with (A) 1.67% (m/v) PL 8–10.5, (B) 2.00% (m/v) PL 8–10.5. Spacer: 42.4 mmol/L L‐Arg, 1.8 mmol/L IDA. Samples contain cIEF gel. Focusing: 25.0 kV, 15.0 min. Cathodic mobilization: 25.0 mmoL/L L‐Asp, pH 10.50, 30.0 kV. All other settings as in Figure 1. Peaks: 1, major variant; 2–4, acidic variants of F(ab´)_2_. “*” indicates residual intact MabThera^®^ after digest.

The p*I* of F(ab´)_2_ was close to intact rituximab. Thus, PL 3–10 was kept at 1.29% (m/v), whereas PL 8–10.5 was increased from 1.00 to 2.50% (m/v) in increments of 0.20–0.30%. 2.00% (m/v) PL 8–10.5 provided best resolution. Three minor acidic variants of F(ab´)_2_ could be addressed, whereas no alkaline fractions were observed (Fig. 3). Mobilization optimization similarly to Section 3.1.1 revealed 25 mmol/L l‐Asp to provide an improved resolution (data not shown). When F(ab´)_2_ concentrations were increased to address minor variants, the durability of the capillary coating was impaired. This is related to the p*I* of F(ab´)_2_ that is even higher than for intact rituximab (Supporting Information Tables S1 and S2). Thus, the tested F(ab´)_2_ concentration was restricted to values below 10 μg/mL, which allowed for a distinction of variants, but still prevented the capillary (coating) from rapid damage. Figure 3 shows a comparison of separation results for the selected PL 8–10.5 contents, i.e., 1.67 and 2.00% (m/v). The resolution of acidic minor variants is progressively improved. A minor peak that is detected 2.0–2.5 min after the major F(ab´)_2_ variant refers to residual intact rituximab since the antibody was apparently not completely digested by IdeS (see “*” in Fig. 3).

#### 3.2.2 CIEF optimization for Fc/2 variants

**Figure 4:**
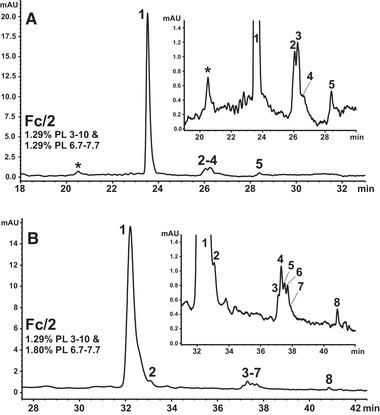
CIEF optimization for variants of Fc/2 fragment derived from MabThera^®^. 1.29% (m/v) PL 3–10 with (A) 1.29% (m/v) PL 6.7–7.7, (B) 1.80% (m/v) PL 6.7–7.7. Spacer: (A) 17.9 mmol/L L‐Arg, (B) 23.2 mmol/L L‐Arg; both 1.8 mmol/L IDA. Samples contain cIEF gel. Focusing: 25.0 kV, for (A) 15.0 min, (B) 30.0 min. Cathodic mobilization: 25.0 mmoL/L L‐Asp, pH 10.50; 30.0 kV. All other settings as in Figure 1. Peaks: 1, major variant; 2–8, acidic variants of Fc/2. “*” indicates a presumed minor alkaline variant, which was not observed in (B) due to the higher UV absorption caused by the increased PL 6.7–7.7 content (see Section 3.2.2).

In pre‐tests, Fc/2 variants were determined to focus in the p*I* domain 7–8 (data not shown). Thus, PL 6.7–7.7 was applied as a narrow pH range CA and was stepwise increased from 1.29 to 2.00% (m/v). The content of PL 3–10 was kept at 1.29% (m/v). With 1.29% (m/v) PL 6.7–7.7, the CIEF profile provided a major peak, a minor cluster of acidic variants (peaks 2–4) and another acidic variant 2.5–3.0 min after the acidic cluster (peak 5). Besides, a minute signal at the alkaline side is present (annotated with an asterisk; all Fig. 4A). When PL 6.7–7.7 was increased to 1.60% (m/v), a shoulder occurred at the alkaline side of the major Fc/2 peak with 15.0 min focusing duration. Increasing the PL 6.7–7.7 content further turned the shoulder into a distinct peak (Supporting Information Fig. S1). Since the entire capillary was filled with sample, the analyte migrated toward its focusing position from the anodic and cathodic side. With the approach toward the p*I*, both the analyte net charge and migration velocity decrease. As the capillary was progressively occupied by PL 6.7–7.7, which is close to the analyte p*I*, this effect became more and more pronounced. This prevented a complete focusing within the selected time interval and led to artificial double peaks (Supporting Information Fig. S1A–C) [56]. This effect was absent in the previous F(ab´)_2_ optimization since the applied narrow pH range CA covered a more extended range of 2.5 pH units. A combination of 1.29% (m/v) PL 3–10 and 1.80% (m/v) PL 6.7–7.7 was selected for the further optimization due to the improved resolution of the acidic cluster (Supporting Information Fig. S1A_1_–C_1_). In order to prevent the artificial peak duplication of the Fc/2 major peak, the focusing duration was increased from 15.0 to 30.0 min. This provided an appropriately focused major Fc/2 peak (Supporting Information Fig. S2A–C) and an improved resolution of the acidic Fc/2 cluster (Supporting Information Fig. S2A_1_–C_1_). A further increase to 35.0 min focusing reduced the resolution of minor variants within the acidic cluster without a beneficial effect on the major peak (data not shown). The optimized CIEF method resolved seven acidic variants from the major Fc/2 peak (Fig. 4B). The small alkaline variant was not detected anymore (Fig. 4A and B) presumably due to the increased UV adsorption caused by the higher PL 6.7–7.7 content.

### 3.3 Determination of p*I* values for intact rituximab and F(ab)_2_ applying optimized CIEF methods

The number of good CA species depends on the covered pH range. This causes deviations of the pH slope from ideal linearity along the formed pH gradient [57, 58]. Thus, p*I* values of proteins should be ideally determined by markers closely flanking the analyte [59], this way minimizing related p*I* biases. In case, one of the flanking markers is focused far away from the analyte, a p*I* determination by extrapolation using two markers focused close‐by either at the acidic or alkaline side of the analyte might constitute a practicable alternative. This is of particular concern, if one of the flanking p*I* markers would be situated outside the pH domain of the narrow pH range CA. Thus, the p*I* for intact MabThera^®^ was extrapolated by markers with p*I* 9.99 and 9.50. The major peak has a p*I* of 9.29, whereas the five acidic variants cover a p*I* range between 9.01 and 9.22. Alkaline variants possess p*I*s up to 9.38 (see Fig. 1B and B_1_ and Supporting Information Table S1).

The p*I* values of the F(ab´)_2_ variants resolved under optimized separation conditions were determined with the same p*I* markers, but by interpolation. This resulted in a p*I* of 9.61 for the major variant and p*I* values between 9.54 and 9.58 for the three resolved acidic variants with a 95% CI ≤0.002 p*I* units (*n* = 3), respectively (Supporting Information Table S2).

### 3.4 Platform approach for Fc/2 variants—Addressing Reditux^TM^ and Humira^®^

**Figure 5:**
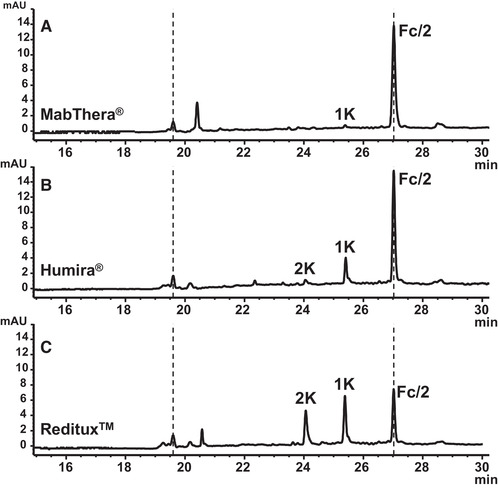
Modified CIEF separation to resolve alkaline Fc/2 variants present in Reditux^TM^ and Humira^®^ (platform approach). 1.29% (m/v) PL 3–10 with 1.03% (m/v) PL 6.7–7.7. Focusing: 25.0 kV, 15.0 min. Spacer: 32.1 mmol/L L‐Arg, 0.4 mmol/L IDA. Cathodic mobilization: 25.0 mmoL/L L‐Asp, pH 10.50; 30.0 kV. Fc/2 fragments of (A) MabThera^®^, (B) Humira^®^, and (C) Reditux^TM^, with 15 μg/mL, respectively. Samples contain cIEF gel. Peaks: 1, major variant; 1K and 2K variants carrying one or two C‐terminal Lys residues. The acidic Fc/2 cluster was present in all tested products, but is not indicated. Electropherograms are time normalized (indicated by dotted lines) for improved comparison.

When the CIEF method optimized for Fc/2 (Section 3.2.2) was applied to Humira^®^ and Reditux^TM^, double peaks were detected at 18 and 23 min, respectively, within the applied 30 min focusing step (Supporting Information Fig. S3). Due to the increased PL 8–10.5 content of the optimized CIEF method, the l‐Arg (cathodic spacer) concentration had to be reduced from 42.9 mmol/L (Section 3.1) to 23.2 mmol/L. Thus, observed double peaks most likely represent alkaline Fc/2 variants. They pass the detection window prior to their complete focusing that takes place behind this window. The double peaks refer to an anodic and cathodic fraction of the Fc/2 variant, respectively, moving toward each other during the focusing step. Several measures were taken to counteract this problem. Primarily, the l‐Arg concentration was increased to 32.1 mmol/L at the expense of a CA reduction to 1.00% (m/v) PL 6.7–7.7. This allowed for an accelerated focusing step of 15.0 min. Under the adjusted CIEF conditions, the postulated alkaline Fc/2 variants were detected during the mobilization step and focused as distinct single peaks well resolved from each other and the major Fc/2 peak (Fig. 5).

Heights of the major Fc/2 variant and of both alkaline variants were similar for Reditux^TM^ (Fig. 5C). In case of Humira^®^, peak heights of the alkaline Fc/2 variants were considerably smaller than the major peak (Fig. 5B). With a t_m_ normalization via two reference peaks, the accordance in the relative focusing position and thus in the p*I* of both alkaline Fc/2 variants could be confirmed for Humira^®^ and Reditux^TM^ (Fig. 5). Moreover, t_m_ normalization even allowed to address a corresponding low abundant alkaline Fc/2 variant in MabThera^®^ otherwise missed (see 1K in Fig. 5A–C). However, the reduction in the PL 6.7–7.7 content resulted in a slightly decreased resolution of the acidic Fc/2 cluster (data not shown).

### 3.5 Determination of p*I* values for Fc/2 variants with adapted platform approach

For the p*I* determination of the Fc/2 variants of MabThera^®^, beside the commercial marker with p*I* 7.00, novel synthetic p*I* markers were applied to comply with the outlined frame conditions. Thus, a marker with p*I* 8.40 (from AES Ltd.), and in‐house synthesized peptides with p*I* 7.56 and 6.77 were applied. Based on their position relative to the Fc/2 variants, p*I* 8.40 and 7.56 were used for the p*I* calculation of the major Fc/2 variant and the acidic cluster. A p*I* of 7.94 was determined for the major Fc/2 peak, whereas the peaks forming the minor acidic cluster were situated between p*I* 7.61 and 7.63. The most acidic Fc/2 variant (peak 8, Fig. 4B) was mobilized together with the marker with p*I* 7.00 (data not shown; Supporting Information Table S2). The p*I* values of the alkaline Fc/2 variants were 8.29 and 8.64 (determined for Reditux^TM^; 95% CI ≤ 0.02 p*I* units; *n* = 3) using markers with p*I* 9.50 and 8.40. For reasons of comparison, the p*I* of the major Fc/2 variant was also determined this way. Its p*I* of 7.86 (95% CI with 0.06 p*I* units; *n* = 3) deviates only by 0.08 p*I* units from the value previously determined with p*I* markers 8.40 and 7.56 for the major Fc/2 variant of MabThera^®^ (Supporting Information Table S2), which legitimates this approach.

### 3.6 Digest with CPB

Prominent alkaline species of Reditux^TM^ showed nearly constant p*I* increments both for Fc/2 and intact mAbs in relation to the major variant, respectively (Supporting Information Tables S1 and S2). Based on the previously described composition of Reditux^TM^ [60, 61], alkaline species present in Humira^®^ and Reditux^TM^ were assumed to represent C‐terminal Lys variants. This hypothesis was tested by digest of the Fc/2 fractions with CPB. CPB selectively cleaves Lys residues from the protein C‐terminus [62, 63]. Both alkaline variants vanished after CPB treatment of Humira^®^ and Reditux^TM^, respectively (Supporting Information Fig. S4), likely proving species with one and two C‐terminal Lys residues. Lys variants addressed by middle‐down CIEF explain prominent alkaline variants of intact Humira^®^ and Reditux^TM^ (Section 3.1.2) and a minor alkaline variant in MabThera^®^ (Section 3.4).

## 4 Concluding remarks

For the first time, CIEF of intact therapeutic mAbs is combined with a corresponding middle‐down approach after digest with IdeS. This allows for a comparison of acidic and alkaline variants on the intact and subunit level. Moreover, an additional dimension can be added to the comparison of similarity between the original reference and follow‐on products on basis of charge variants. For intact MabThera^®^ eight variants (p*I* 9.01–9.38) were resolved. The middle‐down approach resulted in eight Fc/2 variants (p*I* 7.00–7.94) and four F(ab´)_2_ variants (p*I* 9.54–9.61). Despite the p*I* ranges covered by these variants, p*I* differences down to 0.02 units could be distinguished with excellent repeatability (SD < 0.03 p*I* units, respectively). The diverse distribution of p*I*s required the application of peptide markers tailored to focus closely to the respective analytes. Thus, apparent p*I* values are traceable to the applied p*I* markers. In case of MabThera^®^, F(ab´)_2_ is even more alkaline than the intact mAb, whereas Fc/2 is more acidic. The CIEF methods optimized for MabThera^®^ were applied to related products. This approach refers to the industrial need for so‐called platform methods, which are applicable in the analysis of cognate products, i.e., other mAbs, either without modification or after minute refinement. With appropriate knowledge of CIEF fundamentals and a global understanding of the established method(s), this can be achieved in a few steps as shown exemplarily when the optimized Fc/2 method was applied to a copy product of MabThera^®^, i.e., Reditux^TM^, and to adalimumab (Humira^®^). Due to the presence of additional charge variants caused by C‐terminal lysine modifications, the Fc/2 method was adapted based on the settings of the method initially optimized for MabThera^®^ by adapting the alkaline spacer concentration, and a concomitant reduction of the PL 6.7–7.7 content and of the focusing duration.

*The financial support by the Austrian Federal Ministry for Digital and Economic Affairs, the National Foundation of Research, Technology, and Development, and a Start‐up Grant of the Province of Salzburg is gratefully acknowledged. In‐house produced peptide pI markers have been synthesized and characterized by Vesna Stanojlovic and Prof. Chiara Cabrele (both University of Salzburg)*.

*The authors have declared the following competing financial interest(s): Sandoz GmbH and Thermo Fisher Scientific GmbH provided financial support for the Christian Doppler Laboratory for Innovative Tools for Biosimilar Characterization*.

*Dr. Lorenz Stock (former member of the CD‐laboratory) is gratefully acknowledged for his initial CIEF measurements of MabThera^®^ and the adaptation of the IdeS digest protocol*.

## Supporting information

Supplementary Information

**Table S1**. Comparison of pI for variants of intact mAbs, including MabThera®, RedituxTM and Humira® using optimized CIEF conditions and closely flanking pI markers with pI 9.99 and 9.50 (n = 3, respectively). Stated peak numbers refer to Fig. 1B‐D. Distinction between alkaline and acidic variants is due to their relative position to the respective main peak.

**Figure S1**. Progressive peak duplication of Fc/2 due to increased PL6.7‐7.7 content with non‐adjusted focusing duration. 1.29%(m/v) PL3‐10 with (A) 1.60% (m/v) PL 6.7‐7.7, (B) 1.80% (m/v) PL 6.7‐7.7 and (c) 2.00% (m/v) PL 6.7‐7.7. 7.1 μg/mL Fc/2 of MabThera®. All samples contain cIEF gel. Anolyte: 200  mmol/L H_3_PO_4_ (in cIEF gel), catholyte: 300 mmol/L NaOH. Focusing: 25.0 kV, 15.0 min. Spacer: 17.9 mmol/L L‐Arg, 1.8 mmol/L IDA. Cathodic mobilization: 25.0 mmoL/L L‐Asp, pH 10.50. All other settings as in Figure 1. (A1‐C1) depict details of electropherograms (A‐C). Peaks: Fc/2 major variant; *refer to acidic Fc/2 variants addressed previously.
