# Supplementary material for: Intact and middle‐down CIEF of commercial therapeutic monoclonal antibody products under non‐denaturing conditions
Source: Electrophoresis. 2020 Apr 27;41(12):1109–17. doi: 10.1002/elps.202000013 (PMC7317833; doi:10.1002/elps.202000013)
Supplement: Supplementary file 1 — Supplementary Information [file ELPS-41-1109-s001.pdf]

## Supporting information

### Conditioning, rinsing and storage of eCAP$^{TM}$ CIEF capillaries

Prior to their first use, eCAP$^{TM}$ capillaries were rinsed with 10 mmol/L $H_3PO_4$ for 5.0 min and then with ultrapure water for 30.0 min (both with 930 mbar). Before each sample injection, the capillary was rinsed with 10 mmol/L $H_3PO_4$ for 2.0 min and then with ultrapure water for 4.0 min. After each CIEF run the capillary was rinsed with ultrapure water for 4.0 min (with 930 mbar). The capillary has to be prevented from falling dry. Capillaries can be stored for a short period filled with ultrapure water. For storage overnight or over the weekend capillaries were rinsed with cIEF gel for 5.0 min and then stored at +4 °C in this gel with the capillary ends immersed in ultrapure water.

### Digest of mAbs with IdeS

The digest was done by means of a FragIT kit (from Genovis) applicable up to 0.5 mg IgG as outlined by the manufacturer. The FragIT kit contains a spin column for the IdeS digest and a purification column (CaptureSelect) with an affinity ligand, which is selective for the Fc/2 fragments. The latter column was employed in the fractionation of the mAb fragments after the IdeS digest. 50 mmol/L $NH_4HCO_3$ (pH 7.87) was 1+1 mixed with the antibody solution (final mAb concentration was 5 mg/mL) and applied as cleavage and binding buffer. The mAbs were digested for 60 min at room temperature within the spin column with IdeS immobilized on agarose beads. The column eluate was then transferred onto the CaptureSelect column and incubated for 30 min by rotation. $F(ab')_2$ fragments were then eluted by centrifugation before the Fc/2 fraction was detached with a 100 mmol/L glycine solution (pH 3.00) and directly eluted by centrifugation into a vial containing 1.00 mol/L Tris (pH 8.0), which neutralized the eluate. Salt constituents were removed from both eluates by means of Amicon Ultra 0.5 mL centrifugal filter units (from Merck Millipore, Darmstadt, Germany) with a 3 kDa cutoff.
