# Supplementary material for: Intact and middle‐down CIEF of commercial therapeutic monoclonal antibody products under non‐denaturing conditions
Source: Electrophoresis. 2020 Apr 27;41(12):1109–17. doi: 10.1002/elps.202000013 (PMC7317833; doi:10.1002/elps.202000013)
Supplement: Supplementary file 2 — Table S1. Comparison of pI for variants of intact mAbs, including MabThera®, RedituxTM and Humira® using optimized CIEF conditions and closely flanking pI markers with pI 9.99 and 9.50 (n = 3, respectively). Stated peak numbers refer to Fig. 1B‐D. Distinction between alkaline and acidic variants is due to their relative position to the respective main peak. [file ELPS-41-1109-s002.pdf]

Table S1. Comparison of pI for variants of intact mAbs, including MabThera®, Reditux™ and Humira® using optimized CIEF conditions and closely flanking pI markers with pI 9.99 and 9.50 (n=3, respectively). Stated peak numbers refer to Fig. 1B-D. Distinction between alkaline and acidic variants is due to their relative position to the respective main peak.

Antibody	Peak Nr.	Variant	pI ± 95% CI [a]
**MabThera®**			
	1	alkaline 1	9.38±0.006
	2	alkaline 2	n.d. [b]
	3	main	9.29±0.029
	4	acidic 1	9.22±0.055
	5	acidic 2	9.18±0.050
	6	acidic 3	9.12±0.041
	7	acidic 4	9.07±0.063
	8	acidic 5	9.01±0.067
**Reditux™**			
	1	alkaline 1	9.41±0.001
	2	alkaline 2	9.35±0.003
	3	alkaline 3	9.29±0.001
	4	main	9.25±0.002
	5	acidic 1	9.20±0.003
	6	acidic 2	9.16±0.001
	7	acidic 3	9.10±0.004
**Humira®**			
	1	alkaline 1	8.77±0.008
	2	alkaline 2	8.58±0.008
	3	alkaline 3	8.53±0.011
	4	main	8.42±0.010
	5	acidic 1	8.37±0.012
	6	acidic 2	8.29±0.030
	7	acidic 3	8.28±0.019

[a] 95% CI   95% confidence interval (n=3)

[b] not determined

Table S2. Calculated pIs for F(ab´)$_2$ variants and selected Fc/2 variants for MabThera®, as well as for Fc/2 variants for Reditux™. For F(ab´)$_2$ closely flanking pI markers with pI 9.99 and 9.50 were used, whereas for Fc/2 markers with pI 9.50, 8.40, 7.56 and 7.00 were applied. For Fc/2 the modified CIEF platform approach was used for calculation. For a better orientation peak numbers are provided, which refer to Fig. 3B and 4B (for MabThera®) and to Fig. 5C (for Reditux™). 1K and 2K represent Fc/2 variants with one or two additional Lys residues at the C-terminus.

Antibody	Peak Nr.	Variants	pI ± 95% CI [a]
**MabThera®**		**F(ab´)$_2$ [b]**	
	1	main	9.61±0.002
	2	acidic 1	9.58±<0.001
	3	acidic 2	9.56±0.001
	4	acidic 3	9.54±0.001
		**Fc/2 [c]**	
	1	main	7.94±0.015
	3	acidic 2	7.63±0.006
	7	acidic 6	7.61±0.005
	8	acidic 7	7.00 [d]
**Reditux™**		**Fc/2 [e]**	
	2K	alkaline 1	8.64±0.021
	1K	alkaline 2	8.29±0.014
	Fc/2	main	7.86±0.061

[a] 95% CI   95% confidence interval

[b] pI for F(ab´)$_2$ variants of MabThera® determined with pI 9.99 and pI 9.50

[c] pI for Fc/2 variants of MabThera® determined with pI 8.40 and pI 7.56.

[d] Since this variant was mobilized together with pI marker 7.00, no 95% CI is provided.

[e] pI for Fc/2 variants of Reditux™ determined with pI 9.50 and pI 8.40.
