# Supplementary material for: Intact and middle‐down CIEF of commercial therapeutic monoclonal antibody products under non‐denaturing conditions
Source: Electrophoresis. 2020 Apr 27;41(12):1109–17. doi: 10.1002/elps.202000013 (PMC7317833; doi:10.1002/elps.202000013)
Supplement: Supplementary file 3 — Figure S1. Progressive peak duplication of Fc/2 due to increased PL6.7‐7.7 content with non‐adjusted focusing duration. 1.29%(m/v) PL3‐10 with (A) 1.60% (m/v) PL 6.7‐7.7, (B) 1.80% (m/v) PL 6.7‐7.7 and (c) 2.00% (m/v) PL 6.7‐7.7. 7.1 μg/mL Fc/2 of MabThera®. All samples contain cIEF gel. Anolyte: 200 mmol/L H3PO4 (in cIEF gel), catholyte: 300 mmol/L NaOH. Focusing: 25.0 kV, 15.0 min. Spacer: 17.9 mmol/L L‐Arg, 1.8 mmol/L IDA. Cathodic mobilization: 25.0 mmoL/L L‐Asp, pH 10.50. All other settings as in Figure 1. (A1‐C1) depict details of electropherograms (A‐C). Peaks: Fc/2 major variant; *refer to acidic Fc/2 variants addressed previously. [file ELPS-41-1109-s003.pdf]

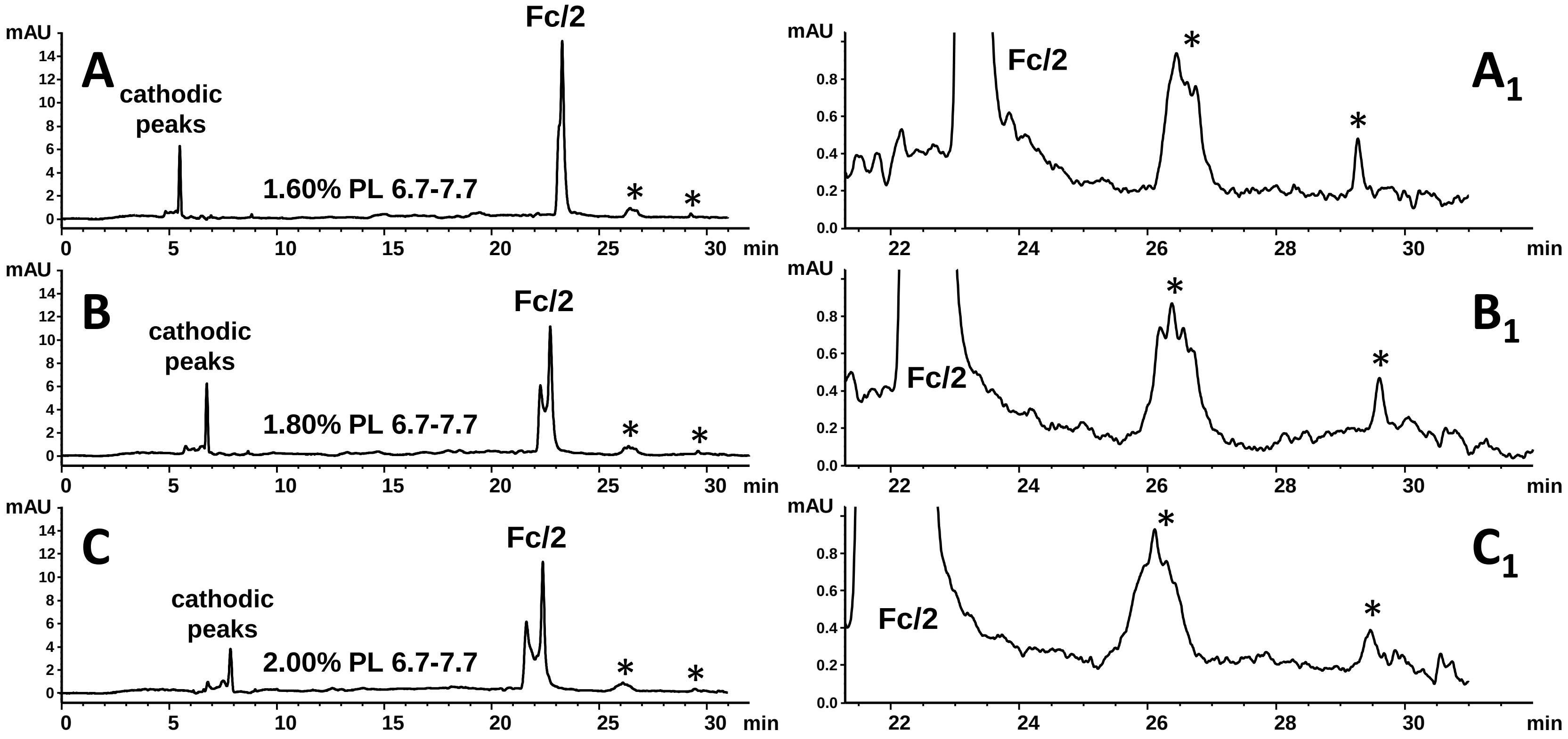

Figure S1. Progressive peak duplication of Fc/2 due to increased PL 6.7-7.7 content with non-adjusted focusing duration. 1.29% (m/v) PL 3-10 with (A) 1.60% (m/v) PL 6.7-7.7, (B) 1.80% (m/v) PL 6.7-7.7 and (c) 2.00% (m/v) PL 6.7-7.7. 7.1 µg/mL Fc/2 of MabThera®. All samples contain cIEF gel. Anolyte: 200 mmol/L H_3PO_4 (in cIEF gel), catholyte: 300 mmol/L NaOH. Focusing: 25.0 kV, 15.0 min. Spacer: 17.9 mmol/L L-Arg, 1.8 mmol/L IDA. Cathodic mobilization: 25.0 mmoL/L L-Asp, pH 10.50. All other settings as in Figure 1. (A_1-C_1) depict details of electropherograms (A-C). Peaks: Fc/2 major variant; * refer to acidic Fc/2 variants addressed previously.

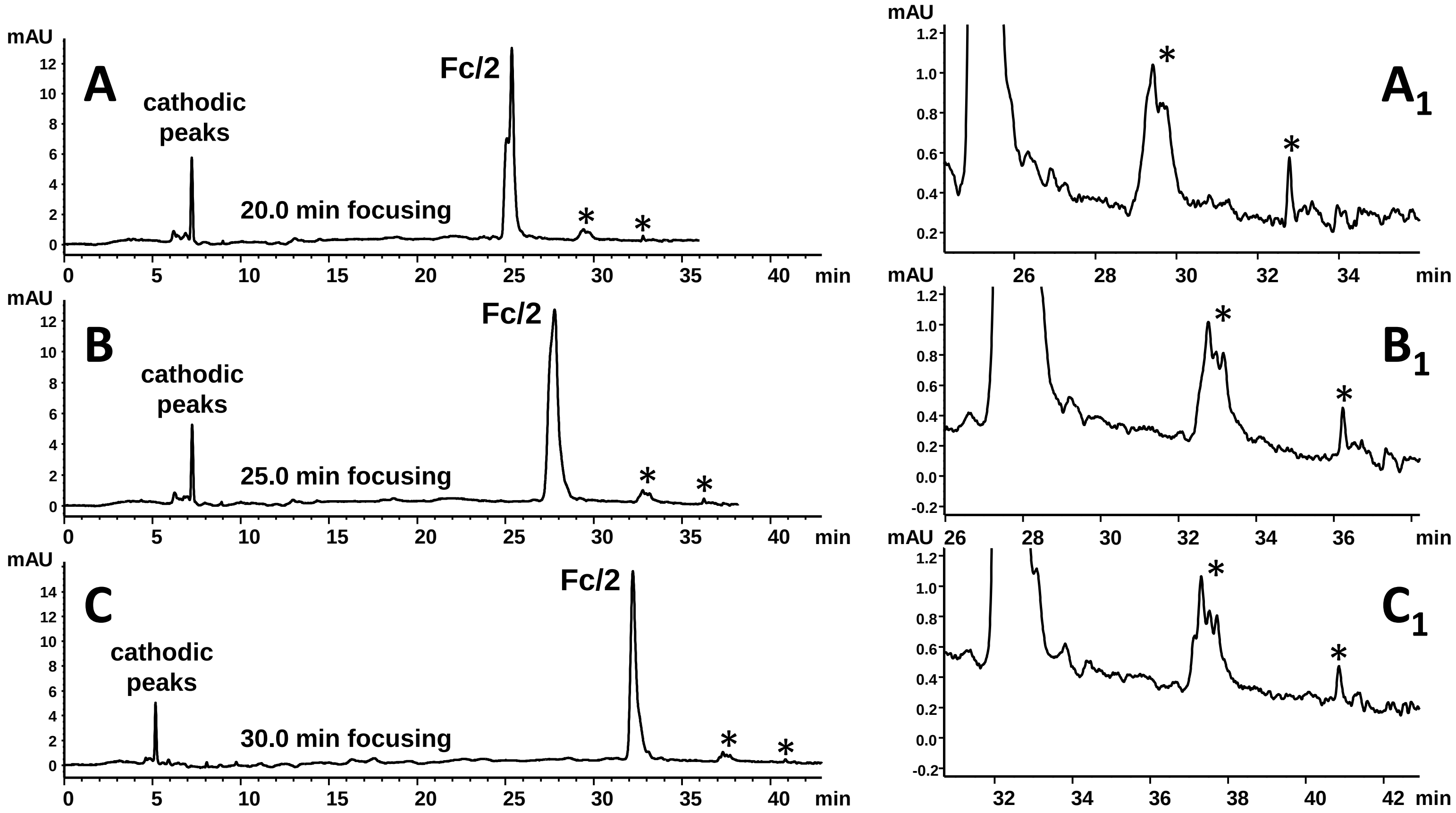

Figure S2. Increase of focusing duration to complete Fc/2 focusing in presence of 1.80% (m/v) PL 6.7-7.7. 1.29% (m/v) PL 3-10 with 1.80% (m/v) PL 6.7-7.7. 7.1 µg/mL Fc/2 of MabThera®. Focusing: 25.0 kV for (A) 20.0 min, (B) 25.0 min, (C) 30.0 min. (A_1-C_1) provide details of minor acidic variants (*). All other settings as in Figure S1.

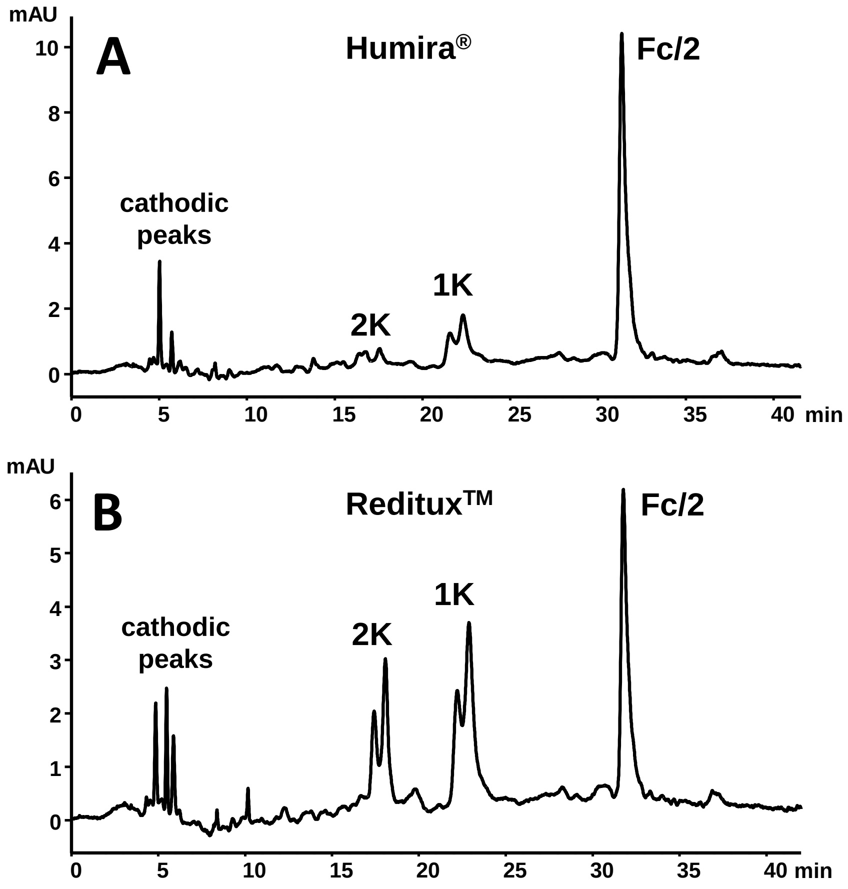

Figure S3. CIEF of Fc/2 variants of (A) Humira® and (B) Reditux™ with settings optimized for MabThera®. 1.29% (m/v) PL 3-10 with 1.80% (m/v) PL 6.7-7.7. Spacer: 23.2 mmol/L L-Arg, 0.4 mmol/L IDA. Focusing: 25.0 kV, 30.0 min. All other settings as in Figure S1. Peaks: 1K = Fc/2 with one C-terminal Lys; 2K = Fc/2 with two C-terminal Lys.

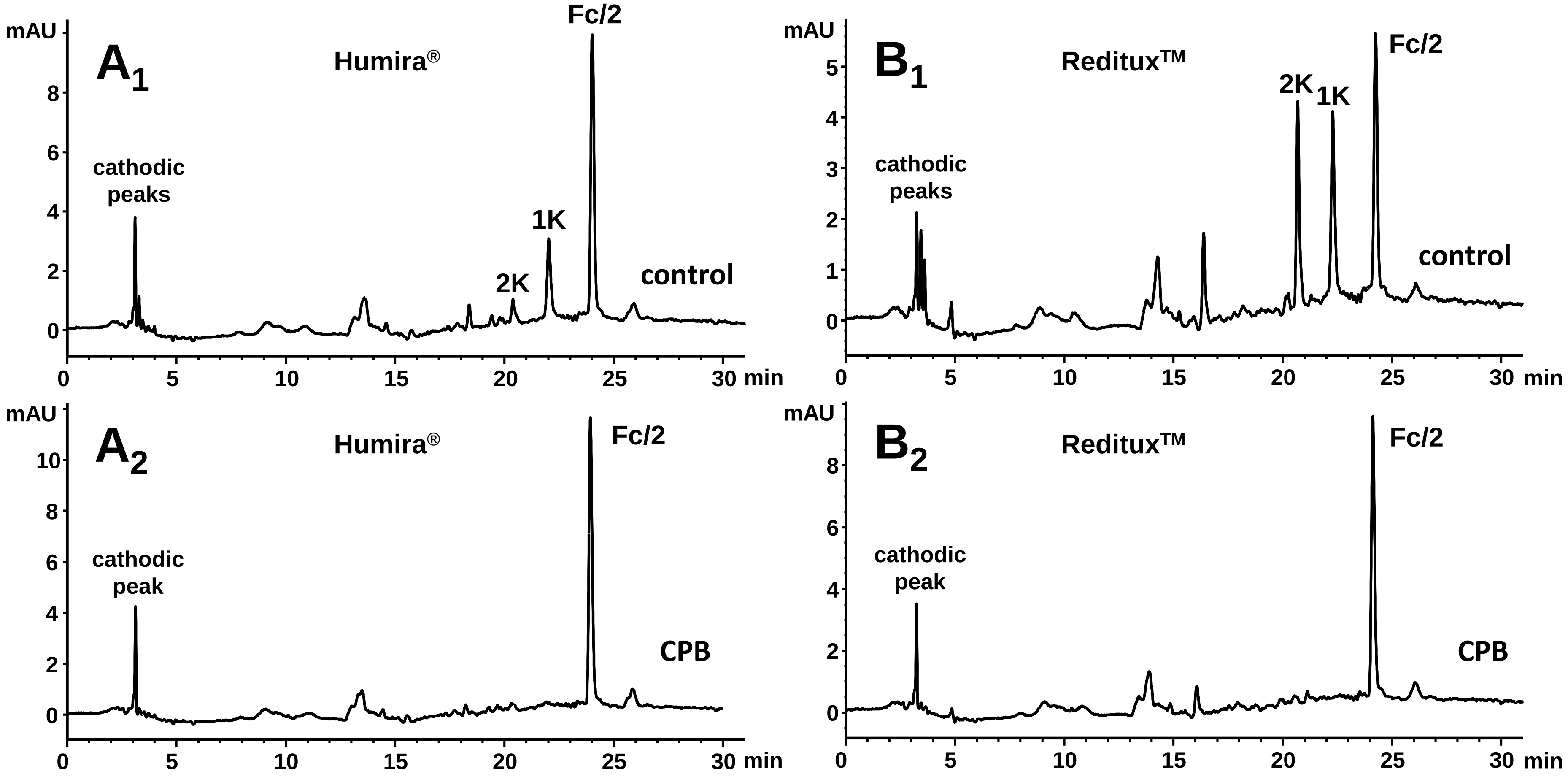

Figure S4. Digest of Fc/2 fragments derived from Humira® and Reditux™ with carboxypeptidase B (CPB). 1.29% (m/v) PL 3-10 with 1.03% (m/v) PL 6.7-7.7. Spacer: 32.1 mmol/L L-Arg, 0.4 mmol/L IDA. Focusing: 25.0 kV, 15.0 min. Fc/2 of (A_1) Humira®, (A_2) Humira® after CPB digest, (B_1) Reditux™, (B_2) Reditux™ after CPB digest. All other settings as in Figure S1.
